# Enhancing the Optically Detected Magnetic Resonance Signal of Organic Molecular Qubits

**DOI:** 10.1021/acscentsci.4c01632

**Published:** 2025-01-03

**Authors:** Yong Rui Poh, Joel Yuen-Zhou

**Affiliations:** Department of Chemistry and Biochemistry, University of California San Diego, La Jolla, California 92093, United States

## Abstract

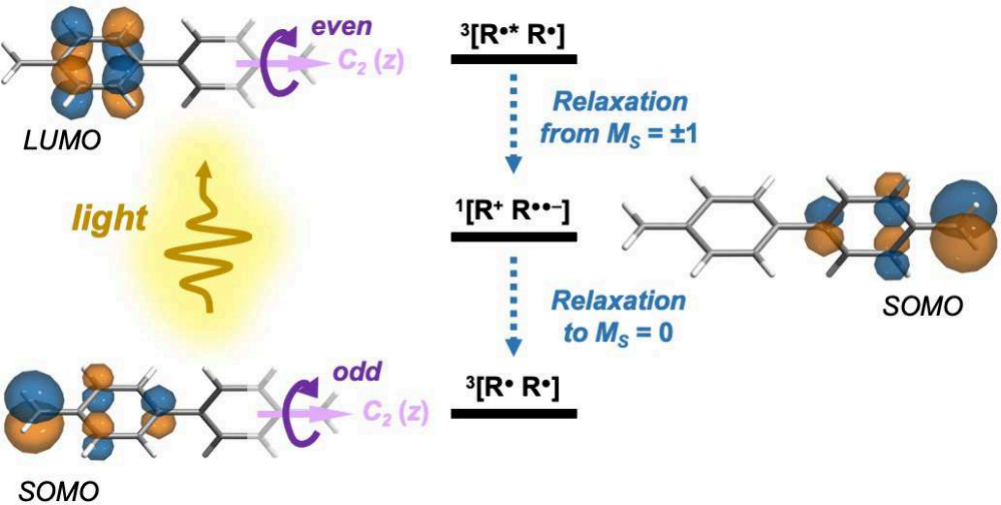

In quantum information science and sensing, electron spins are often purified into a specific polarization through an optical-spin interface, a process known as optically detected magnetic resonance (ODMR). Diamond-NV centers and transition metals are both excellent platforms for these so-called color centers, while metal-free molecular analogues are also gaining popularity for their extended polarization lifetimes, milder environmental impacts, and reduced costs. In our earlier attempt at designing such organic high-spin π-diradicals, we proposed to spin-polarize by shelving triplet *M*_*S*_ = ±1 populations as singlets. This was recently verified by experiments albeit with low ODMR contrasts of <1% at temperatures above 5 K. In this work, we propose to improve the ODMR signal by moving singlet populations back into the triplet *M*_*S*_ = 0 sublevel, designing a true carbon-based molecular analogue to the NV center. Our proposal is based upon transition-orbital and group-theoretical analyses of beyond-nearest-neighbor spin–orbit couplings, which are further confirmed by ab initio calculations of a realistic trityl-based radical dimer. Microkinetic analyses point toward high ODMR contrasts of around 30% under experimentally feasible conditions, a stark improvement from previous works. Finally, in our quest toward ground-state optically addressable molecular spin qubits, we exemplify how our symmetry-based design avoids Zeeman-induced singlet–triplet mixings, setting the scene for realizing electron spin qubit gates.

## Abstract 2

We propose organic π-diradicals that, upon photoexcitation, will relax through two consecutive spin-changing steps. This aligns the radical spins, observed as a change in the emission signal.

## Introduction

**Figure 1:**
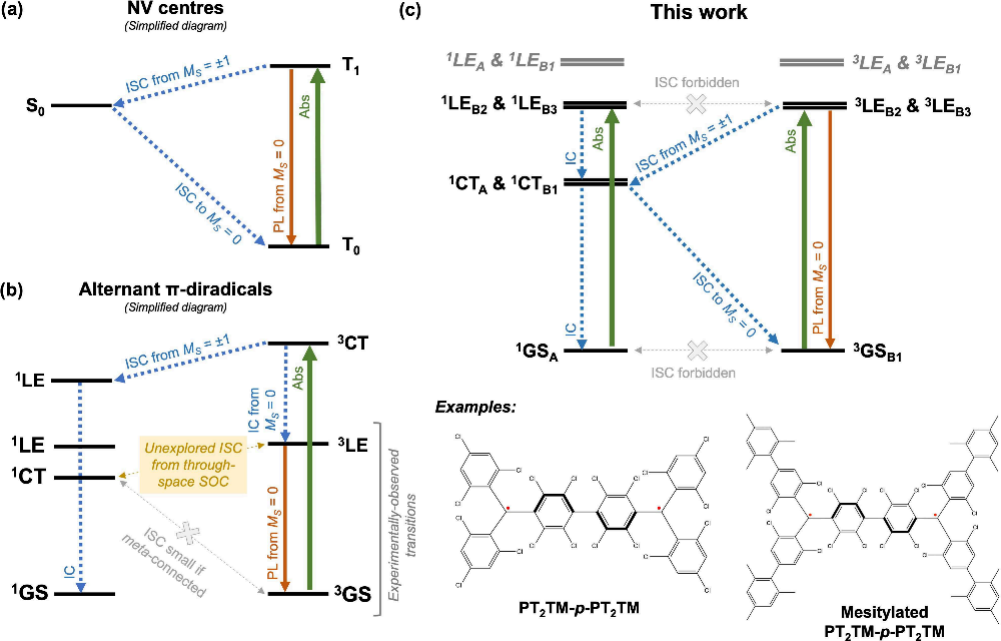
(a) Simplified ODMR mechanism typical of diamond-NV defects. (b) Simplified ODMR mechanism of alternant π-diradicals as explored by our previous work.^52^ Note that the CT-to-GS ISC rates are small if the two benzylic radicals are tethered at their *meta* positions, which is avoided by our new design (see main text). (c) ODMR mechanism of our new design, which more closely resembles the spin polarization pathway of NV centers. Also drawn are the PT_2_TM-*p*-PT_2_TM diradical and its mesitylated counterpart, both of which satisfy our design requirements. (Abbreviations of electronic transitions: Abs = absorption; PL = photoluminescence; IC = internal conversion; ISC = intersystem crossing. Labels for electronic states: GS = ground state; LE = local excitation; CT = charge transfer. These labels are inherited from our previous analysis of the π-diradical electronic structure^52^ with subscripts *A*, *B*_1_, *B*_2_, and *B*_3_ signifying the state irreps.)

Polarized electron spins are promising quantum bit (qubit) candidates^1^ with applications in quantum sensing^2^ and quantum information science.^3^ Purifying these spin magnetic dipoles into a particular polarization requires irreversible decays and these have been achieved in solid-state spin defects^4−13^ by coupling the microwave-addressable electron spins with their orbital degrees of freedom [Figure 1a]. Because the latter lies high in the optical regime, this technique has become known as optically detected magnetic resonance (ODMR) – “detected” because the same optical-spin interface also enables readout of the spin polarization. Platforms hosting optically addressable spins are then referred to as color centers.

While early endeavors in this direction have focused on solid-state defects such as nitrogen-vacancy (NV) centers in diamond,^14^ their poor scalability and tunability have motivated the community to shift toward molecular platforms.^15−24^ Transition metal complexes with high-spin ground states (GSs) offer the most natural starting point and this has been successful in Cr(IV), V(III), Ni(II), and, most recently, Ir(IV) complexes.^25−37^ However, in none of these systems were the spin lifetimes as long as NV centers, essential for applications like qubit operations, and this has been attributed to the larger spin–orbit couplings (SOCs) introduced by metals. Other challenges include higher costs and poorer sustainability. This has prompted a search for a molecular NV-center mimic that is metal-free, just like diamond itself, notwithstanding the benefits of metal-based qubit systems such as higher biocompatibility^38,39^ and simpler protocols for multiqubit addressability.^16,40−42^

As a first step along this path, organic molecules were designed to demonstrate ODMR in high-spin excited states while acknowledging the limited lifetimes of these electronic excitations.^43−51^ Subsequently, our group theoretically proposed a class of organic π-diradicals with optically addressable high-spin ground states^52^ that were also experimentally validated by Chowdhury et al.^53^ and Kopp et al.^54^ In all of these systems, spin polarization was likely attained by shelving the triplet *M*_*S*_ = ±1 population in the singlet manifold via spin-selective intersystem crossings (ISCs) while utilizing the presence of additional singlet charge-recombined states absent from the triplet manifold [Figure 1b]. (Interestingly, such imbalances have recently been proposed as evidence of low-energy electronic spin isomers.^55^) Missing from the ODMR cycles are the ISCs between singlets and triplets selective for the *M*_*S*_ = 0 triplet sublevel, which the NV center exhibits. As mentioned in our previous work,^52^ this limits the maximum possible triplet *M*_*S*_ = 0 population to 25% at steady state, constraining the ODMR resolution. Moreover, this increases the risk of metastable singlet molecules returning as *M*_*S*_ = ±1 triplets, which would negate the accumulated spin polarization. Indeed, in the aforementioned experimental works, poor ODMR contrasts of <1% were observed at temperatures above 5 K. We note in passing that, apart from π-diradicals, alkaline earth metal complexes^56,57^ and nitrenes^58^ also display diradical properties and their functions as molecular color centers have not been fully explored.

Our earlier theoretical model^52^ had focused primarily on nearest-neighbor-only (through-bond-only) SOCs, which predicted ISCs to have Δ*M*_*S*_ = 0 (±1) selectivity for electron spins quantized parallelly (perpendicularly) to the dimer linkage. However, beyond-nearest-neighbor SOC effects have been shown to be experimentally relevant in ODMR, as in the recent work by Kopp et al.^54^ [Figure 1b], and such effects can induce ISCs with an opposite spin selectivity. Motivated by this observation, in this work, we design another class of organic π-diradicals, this time capable of ISCs involving both triplet *M*_*S*_ = ±1 and *M*_*S*_ = 0 sublevels of *different* electronic states as facilitated by beyond-nearest-neighbor and nearest-neighbor SOCs, respectively [Figure 1c]. This way, we create a true analogue of the NV center, which we predict to demonstrate enhanced ODMR signals by optically pumping the near-degenerate triplet and singlet local excitations. Our design principle stems from a careful group-theoretical analysis of the transitioning orbitals, which coincidentally also solves the problem of singlet–triplet mixing during electron paramagnetic resonance (EPR) measurements of weakly coupled diradicals (as most π-diradicals are^54,59^). As a realistic prototypical example of our design, the electronic structure of PT_2_TM-*p*-PT_2_TM [Figure 1c] was shown at both density functional theory (DFT) and multiconfigurational (MC) levels of theory to demonstrate an ODMR mechanism similar to the NV center. The potential for signal enhancement through this ODMR pathway is supported by microkinetic analyses, where we found high ODMR contrasts of around 30% to be experimentally possible (as compared to <1% in earlier theoretical^52^ and experimental^53,54^ studies). In this regime, the steady-state triplet population exceeds 25%, which is the upper bound of prior works.^52−54^ While not explored by the present work, we expect further mesitylation [Figure 1c] to improve the luminescence quantum yield via geometrical relaxation,^60,61^ as exemplified by Chowdhury et al.^53^ to produce near-unity photoluminescence yields. This paves the way toward better designs of optically addressable organic spins. Note that the alternacy symmetry of PT_2_TM-*p*-PT_2_TM is not important to our design, which should be separated from our earlier work.^52^

## Results and Discussion

### Investigating the Role of Beyond-Nearest-Neighbor SOCs

**Figure 2:**
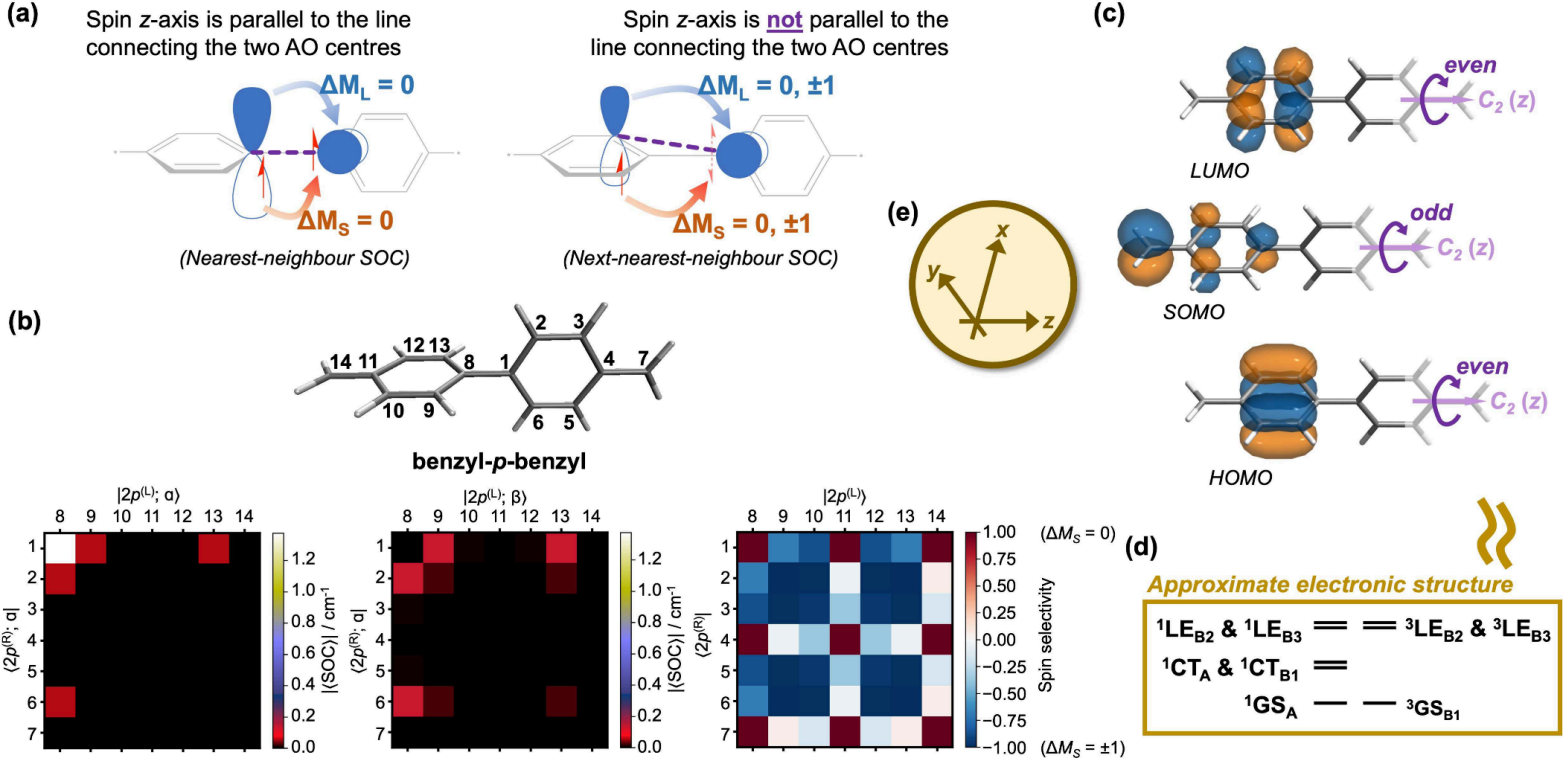
(a) Breakdown of the spin selection rule for ISCs involving next-nearest-neighbor SOCs. (b) Amplitudes of the SOC matrix elements evaluated between two carbon 2*p* AOs centered on opposite π-systems of the benzyl-*p*-benzyl diradical. Each 2*p* AO is assumed to be occupied by one full electron with either the same or opposite spin as its counterpart on the other benzylic fragment. Taking the difference between the matrix elements-squared of the two spin alignments and weighing the result by their sums give the spin selectivity. (c) Sketches of the frontier Hückel MOs for the benzylic monoradical. Also illustrated are their parities under *C*_2_(*z*) rotation. (d) Approximate electronic structure of benzyl-*p*-benzyl obtained from the Hückel theory analysis in (c). The excitation labels are inherited from ref (52) and the subscripts indicate the state irreps. In this work, all molecular geometries are presented in the coordinate frame depicted in (e).

As we have previously shown,^52^ when two benzylic radicals are covalently connected with a significant torsion (as is the way most π-diradicals are experimentally constructed^59,62−78^), its SOC matrix elements are dominated by nearest-neighbor interactions between the two 2*p* atomic orbitals (AOs) of opposite π-rings (reason: El-Sayed rule^1^) that are closest to the dimer linkage (reason: SOC is a local effect). Henceforth, we refer solely to π AOs. Furthermore, if the spin quantisation axis lies parallel (perpendicular) to the line connecting the two 2*p* AO centers, then the ISC spin selectivity arising from the SOC matrix elements is strictly Δ*M*_*S*_ = 0 (±1) due to symmetry.^52^ Note that the last condition is satisfied for interactions at the dimer bond because the spin axes (wihout applying any magnetic fields) coincide with the dimer’s high symmetry axes. However, ISCs in π-diradicals occur not between pairs of 2*p* AOs but between pairs of *delocalized* molecular orbitals (MOs) that are each a linear combination of a few 2*p* AOs. When one of the transitioning MOs contains a node at the dimer linkage, the relevant SOC matrix elements necessarily involve next-nearest-neighbor couplings not considered by the above analysis. Such effects are generally small following the local nature of SOCs, yet they can be appreciable if most of the transitioning MO’s amplitude is piled up at these next-nearest-neighbor sites (as opposed to nearest-neighbor couplings weighted by small MO amplitudes). Importantly, next-nearest-neighbor interactions involve two 2*p* AO centers that are not colinear with the spin quantisation axis, causing the ISC selectivity to break down [Figure 2a]. Indeed, for two benzylic radicals covalently tethered at the *para* positions with 90° torsion (hereafter called benzyl-*p*-benzyl), the next-nearest-neighbor SOC evaluated over singly occupied 2*p* AOs biases the Δ*M*_*S*_ = ±1 transitions with 68.1% selectivity (noting that the spin quantisation (*z*-)axis lies parallel to the dimer linkage due to the molecular *D*_2*d*_ point group symmetry) and this SOC has a significant amplitude of 0.13 cm^–1^ [Figure 2b]. For comparsion, the nearest-neighbor analogue has a *perfect* spin selectivity of Δ*M*_*S*_ = 0 with a larger amplitude of 1.37 cm^–1^. Note that these SOC matrix elements represent upper bounds to the true molecular values because we have assumed one full electron in each AO.

### Perfecting the Spin Selectivity Using the Molecular Point Group Symmetry

That ISC in π-diradicals can actually occur with both spin selectivities is contrary to most theoretical analyses^80−82^ (including ours^52^) and opens up the possibility of transferring the full electronic structure of NV centers into molecules. Our next step is to perfect the next-nearest-neighbor spin selectivity via symmetry considerations. We note that by connecting two benzylic radicals at their *para* positions, the resulting diradical has at least a *D*_2_ point group symmetry with irreducible representations (irreps) organized by their characters under *C*_2_(*y*) and *C*_2_(*z*) rotations. The irrep of any electronic state is in turn the direct product of its orbital and spin irreps. Focusing on the orbital part, if we continue to align the *z*-axis along the dimer linkage [Figure 2b,e], then a *C*_2_(*y*) rotation becomes equivalent to swapping the two monomers. Therefore, for two weakly interacting π-systems of significant torsion, the low-lying excitations are expected to appear in near-degenerate pairs of {*A*, *B*_1_} and {*B*_2_, *B*_3_}, each comprising a state of opposite parity under *C*_2_(*y*), i.e., equal probability of exciting either monomer. These two pairs can be distinguished by their transformations under *C*_2_(*z*) and, for that, it is easier to think in the basis of *monomeric π* MOs. We first note that 2*p* AOs have π symmetry, that is, their phases change under *C*_2_ rotation about the AO center. Further, for the two 2*p* AOs at the dimer linkage, that π-symmetry-defining *C*_2_ rotation coincides with the diradical’s *C*_2_(*z*) symmetry element. Hence, a monomeric MO with a nonzero amplitude at the dimer linkage *must* be antisymmetric under *C*_2_(*z*) rotation. By contrast, for a monomeric MO to be symmetric under *C*_2_(*z*) rotation, it *must* possess a node at the dimer linkage. For instance, the Hückel method predicts the highest occupied, singly occupied and lowest unoccupied MOs (abbreviated as HOMO, SOMO, and LUMO, respectively) of the benzylic radical to have +1, −1, and +1 characters under *C*_2_(*z*) rotation [Figure 2c]. Finally, we recall that the symmetry of the orbital wave function is the direct product of the symmetries of all singly occupied orbitals. Therefore, the ground states of benzyl-*p*-benzyl, having two unpaired electrons in the SOMOs, transform as either *A* or *B*_1_. Similarly, the lowest charge transfer (CT) singlets with all doubly occupied orbitals (also known as charge-recombined or zwitterionic states of the two radicals) also transform as either *A* or *B*_1_. As for the lowest local excitations (LEs), they are a linear combination of HOMO-to-SOMO and SOMO-to-LUMO excitations (an outcome of alternancy symmetry; see ref (52)) and hence transform as either *B*_2_ or *B*_3_. Including the characters under *C*_2_(*y*) rotation yields the approximate energy level diagram illustrated in Figure 2d (for a review of the electronic structure of benzylic diradicals, see our earlier work^52^).

Returning to our goal of attaining perfect ISC spin selectivities, we note that the singlet, triplet *M*_*S*_ = 0, and triplet *M*_*S*_ = ±1 spin sublevels transform as *A*, *B*_1_, and {*B*_2_, *B*_3_}, respectively.^83,84^ For an ISC to be symmetry-allowed, the SOC matrix elements must contain the totally symmetric irrep. Hence, because the SOC Hamiltonian is totally symmetric, ISC processes in benzyl-*p*-benzyl are spin-selective for Δ*M*_*S*_ = ±1 when moving between triplet LEs and singlet CTs, and Δ*M*_*S*_ = 0 when moving between singlet CTs and triplet GSs. Actually, we already knew this from the previous section: The first ISC occurs between SOMOs and LUMOs (or HOMOs) of opposite monomers and only the latter has a node at the dimer linkage [Figure 2c]. Thus, this process occurs predominantly via next-nearest-neighbor SOCs, which we found before to exhibit Δ*M*_*S*_ = ±1 selectivity [Figure 2b]. Similarly, the second ISC connects between two SOMOs of opposite monomers and is mediated mostly by nearest-neighbor SOCs with Δ*M*_*S*_ = 0 selectivity [Figure 2b]. Yet, the key improvement achieved by this section is the perfection of ISC spin selectivities to 100% by making the opposite spin channel symmetry-forbidden. From a microscopic perspective, we have destructively interfered any deleterious ISC channels using other higher-nearest-neighbor SOCs, treated more generally by the group-theoretical approach. Importantly, this electronic structure produces the ODMR mechanism shown in Figure 1c, where populations in the triplet *M*_*S*_ = ±1 LEs are moved into the triplet *M*_*S*_ = 0 GS via two sequential ISCs of opposite spin selectivities. In other words, such molecules exhibit the ODMR mechanism of NV centers.

As an aside, while it is tempting to consider only symmetry arguments and ignore the discussions made in the previous section, note that symmetry-allowed transitions need not occur with high amplitudes. Therefore, one still needs to check if the allowed ISCs are facilitated by either nearest- or next-nearest-neighbor SOCs, which have the largest amplitudes. For example, the *meta*-connected diradicals explored in our previous work^52^ have negligible SOCs between singlet CTs and triplet GSs [Figure 1b] because they are dominated by third-nearest-neighbor effects with a maximum SOC amplitude of 0.02 cm^–1^ [Figure 2b]. In other words, our plan for enhanced ODMR contrasts requires *para*-connectivity between the two benzylic radicals, consistent with our group-theoretical analysis (*meta*-connectivity does not yield *D*_2_ point group symmetry). We note in passing that vibronic perturbations to the SOC operator, which are not considered by this study, can modify the spin selectivity as well.^13,85^ This will be explored in our upcoming studies.

### Our Proposal

We now introduce a molecular design that realizes the ODMR mechanism described in Figure 1c:

1. **The π-diradical should be a dimer centralized around a benzyl-*****p*****-benzyl-like framework.** This ensures that the diradical’s electronic structure resembles Figure 2d.
2. **Sufficient steric hindrances should be installed around the dimer linkage to maintain a 90° torsion.** Otherwise, *para* connectivity facilitates π-bonding and encourages a singlet ground state.^66,86−89^ For instance, chloro substituents can be introduced at the *meta* positions, which is common practice when synthesizing luminescent benzyl-type radicals.^66^
3. **Any additional π-conjugation to the benzylic radical should continue to localize the lowest-lying excitations around the benzylic group.** For instance, phenyl substituents added to the benzylic radical (typically used to improve radical stability and luminescence) should have less-extensive internal π-conjugation so that their lowest transitions are energetically separated from the benzylic fragment’s.
4. **These extra substituents should also maintain the monoradical’s*C*_2_(*z*) symmetry, where the*z*-axis lies along the*ipso*-*para*direction.** The reason is that the *C*_2_(*z*) axis is the *only* symmetry element discriminating between the monomeric π MOs [Figure 2c], thus their parities under *C*_2_(*z*) dictate the spin selectivity of their intermonomer ISCs. (For the same reason, diradicals of *D*_2_ point group symmetry are not the only molecules satisfying our design criteria. Nondimeric diradicals can also be constructed with *C*_2_ symmetry and the above near-degenerate pairs of {*A*, *B*_1_} and {*B*_2_, *B*_3_} excitations will become split in energy, thereby offering a ladder of states through which the singlet molecules can decay. Here, we will focus solely on dimeric diradicals of *D*_2_ symmetry for simplicity.) As an example, when substituting the two hydrogens on the benzylic center, both hydrogens should be replaced simultaneously and with identical moieties.

**Figure 3:**
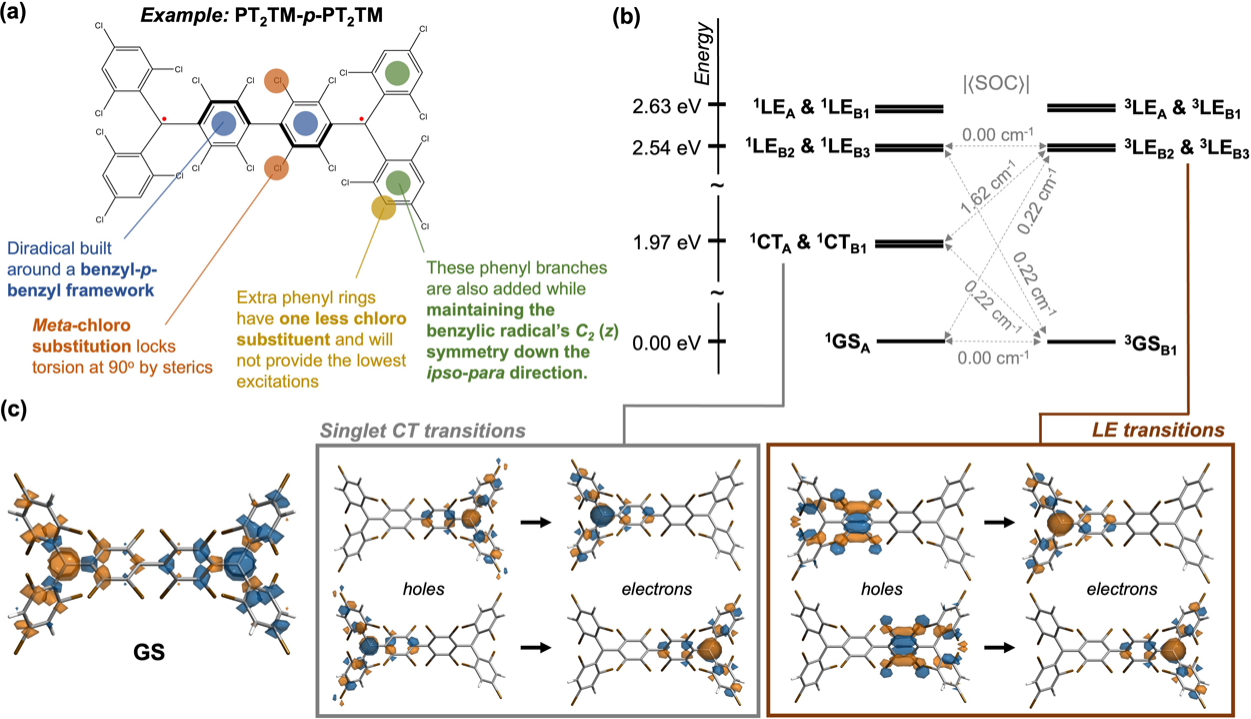
(a) Features of our diradical design, demonstrated on the PT_2_TM-*p*-PT_2_TM diradical. (b) Energy level diagram of PT_2_TM-*p*-PT_2_TM, computed using TDDFT. Also included are the root-mean-squared SOC matrix elements, averaged over all channels. These calculations were performed using MCSCF/CI methods on the central tetra-chlorinated benzyl-*p*-benzyl fragment. (c) Ground state spin density (isovalue = 0.004) and natural transition orbitals (isovalue = 0.040) for the first few excited states of PT_2_TM-*p*-PT_2_TM, obtained using BS-DFT/TDDFT.

These features are summarized in Figure 3a using our prototype PT_2_TM-*p*-PT_2_TM diradical as an example. Note that its alternacy symmetry is not crucial to our design, which should be distinguished from our earlier work.^52^

### Ab Initio Calculations of the Prototype PT_2_TM-*p*-PT_2_TM Diradical

To support the above theory, calculations were performed on the PT_2_TM-*p*-PT_2_TM diradical as a representative example of experimentally feasible diradicals. Starting with its ground state properties, the triplet equilibrium geometry was found using unrestricted DFT to display a significant torsion of 86.4° around the dimer linkage. At this geometry, the ground singlet state is only 1.08 meV lower in energy (equivalent to thermal energy at 12.5 K) than the ground triplet state, computed using broken-symmetry (BS) DFT. Thus, the *m*-chloro substituents sufficiently minimize π-bonding between the two radicals, that is, we have obtained a true diradical.

Excited state properties were then estimated using unrestricted time-dependent DFT (TDDFT) within the Tamm–Dancoff approximation (TDA). The electronic structure fits our requirements for the ODMR mechanism [Figure 1c] with the exception of another low-lying LE pair of irreps *A* and *B*_1_ at around 0.1 eV away from the lowest LEs [Figure 3b]. As eluded in Figure 1c, these excitations are spectroscopically distinguishable from the lowest LEs by wavelength and may be avoided by optically pumping with a single-frequency laser. Otherwise, there is a possibility of avoiding these excitations by aligning the excitation polarization along the lowest LEs’ transition dipole moments, i.e., along the *xy*-plane. *Even if these undesired LEs were photoexcited,* any deleterious Δ*M*_*S*_ = 0 ISCs to the lowest singlet CTs are expected to be slower than internal conversions (ICs) to the lowest LEs due to Kasha’s rule^90^ and will have a minimal impact on the proposed ODMR pathway. Further verification of this energy gap was achieved by applying multiconfigurational self-consistent field (MCSCF) and configuration interaction (CI) methods on the monoradical. Here, the dimer linkage was capped with a hydrogen substituent. This approach places the energy separation between the two lowest LEs at 0.21 eV, consistent with the TDDFT/TDA predictions [Figure 3b].

As speculated, the lowest LE transitions are largely localized on the central (tetra-chlorinated) benzyl-*p*-benzyl fragment [Figure 3c]. Hence, for computational efficiency, the SOC matrix elements were computed for this central fragment instead, capping any open ends with hydrogen. Indeed, the MCSCF/CI results suggest appreciable SOC matrix elements along the proposed ODMR pathway [Figure 3b]. While, strictly speaking, these calculations present only an upper bound to the true SOC matrix elements of the full PT_2_TM-*p*-PT_2_TM diradical, their values should not decrease by much upon weak π-conjugation to the additional trichlorophenyl substituents. Most importantly, these calculations lend credence to the theoretical proposal presented by this work.

### Symmetry Avoids Zeeman-Induced Singlet–Triplet Mixings during EPR Spectroscopy

Interestingly, in connecting two identical monoradicals of (at least) *C*_2_ point group symmetry to form a diradical of (at least) *D*_2_ point group symmetry, the resulting structure can avoid undesirable singlet–triplet mixings found in many weakly coupled diradicals under an applied magnetic field. This would alleviate most magnetic-field-induced decoherence events in the ground state^91,92^ (notice that the singlet and triplet GSs differ by either the coherence or a single spin flip^52^). It also facilitates Rabi nutation experiments in EPR spectroscopy,^54^ among other benefits.

To introduce the problem, we consider the spin Hamiltonian *Ĥ*_spin_ of two electron spins **Ŝ**_*j*_ (*j* = 1, 2) under an applied magnetic field **B**:
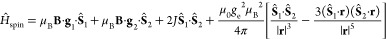
1The first two terms represent each spin’s interaction with the magnetic field (i.e., the spin Zeeman terms), characterized by different *g*-tensors **g**_*j*_ (*j* = 1, 2). The third term symbolizes the exchange interaction between the two spins with coupling 2*J* and is responsible for singlet–triplet splittings. The last term denotes the spin–spin dipolar interaction that gives rise to zero-field splittings in organic diradicals; here, the spatial degrees of freedom (characterized by **r**) have been integrated over some orbital subspace (for instance, the ground state). Finally, the symbols μ_B_, μ_0_, and *g*_e_ represent, respectively, the Bohr magneton, the vacuum magnetic permeability, and the electron *g*-factor. Implicit to the above expression is the definition of a four-dimensional Hilbert space spanned by tensor products of the two electron spin states. By applying the angular momentum sum rules, one may also express the Hilbert space in the basis of one singlet and three triplet spin states.^91,93^ This amounts to re-expressing eq 1 in terms of the total spin operator **Ŝ** ≡ **Ŝ**_1_ + **Ŝ**_2_, which yields
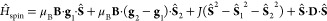
2The procedure for transforming the last term of eq 1 into the last term of eq 2 is presented in most EPR texts^91,92^ with **D** being referred to as the zero-field splitting tensor. We now see the problem: If **g**_1_ ≠ **g**_2_, true in most organic systems, then the singlet–triplet basis block-diagonalizes every term in eq 2 (with the triplets forming their own block) except for the second one. In other words, the second term induces mixings between the singlet state and the triplet manifold. This, as mentioned, leads to deleterious effects such as spin decoherence^91,92^ and additional Rabi frequencies.^54^ One solution would be to engineer an exchange coupling *J* that is large enough for the Zeeman-induced singlet–triplet couplings of ∼μ_B_*B*|*g*_2_ – *g*_1_| to be a perturbation relative to the singlet–triplet gap. However, as explained in our earlier work,^52^ further enhancement of the exchange coupling in π-diradicals is likely to result in a singlet GS, i.e., *J* is typically antiferromagnetic.^66,86−88^

The alternative approach would be to somehow make elements that differ between **g**_1_ and **g**_2_ disappear under experimental conditions. This can be achieved in our diradicals by aligning the magnetic field **B** parallelly to the dimer linkage. (We acknowledge that aligning the molecules, say, in a liquid crystal, may be an experimental challenge, although this has been previously demonstrated.^94,95^) To see that, we first note that elements of the *g*-tensor have the following approximate expression, obtained from second-order perturbation theory in the SOC:^91,92^
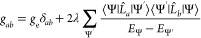
3Here, λ is an effective SOC constant and {|Ψ*′*⟩} and {*E*_Ψ*′*_} respectively denote the orbital eigenfunctions and eigenenergies of the molecule’s nonrelativistic electronic Hamiltonian, with Ψ being the orbital eigenfunction under consideration (say, the ground state wave function). The indices *a* and *b* run over all three-dimensional coordinates (such as *x*, *y*, and *z*) and *L̂*_*a*_ is the *a*-th component of the orbital angular momentum operator **L̂**. Because each monoradical of our proposed system is symmetric about *C*_2_(*z*) rotation (using the coordinate frame defined by Figure 2e, i.e., *z*-axis along dimer linkage), the *g*-tensor must remain invariant under *C*_2_(*z*) rotation. Such a rotation takes {*L̂*_*x*_, *L̂*_*y*_, *L̂*_*z*_} → {−*L̂*_*x*_, −*L̂*_*y*_, *L̂*_*z*_}, hence, using eq 3, we obtain
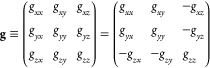
4By contradiction, it must be that
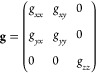
5Next, the two monoradicals in the dimer are related by a *C*_2_(*y*) rotation. Taking eq 5 to be the *g*-tensor of radical 1, we find the *g*-tensors of both radicals to be
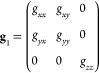
6
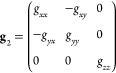
7Therefore, because both **g**_1_ and **g**_2_ have the same *g*_*zz*_ components, the second term of eq 2 vanishes when the magnetic field **B** falls along the *z*-axis (the dimer linkage), i.e.,
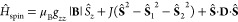
8The singlet–triplet states may now block-diagonalize *Ĥ*_spin_ with no singlet–triplet mixings present. Furthermore, due to the approximate *D*_2*d*_ point group symmetry of our system, the zero-field splitting caused by **D** also occurs along the *z*-axis. This may be further rationalized by the cylindrical spin density distribution of the diradical.^92^ Therefore, the singlet–triplet basis fully diagonalizes *Ĥ*_spin_ in eq 8 when the magnetic field is applied along the dimer linkage.

### Predicted ODMR Contrasts

As a final theoretical piece to this puzzle, we construct a microkinetic model of the excited state kinetics and predict the ODMR contrast at steady state. For simplicity, we shall take the incoherent limit and consider only changes to the eigenstate populations. Also, populations of degenerate eigenstates are pooled into a single variable. Therefore, the relevant eigenstates are

a. the singlet GS (labeled GS, degeneracy = 1)
b. the triplet *M*_*S*_ = 0 GS (labeled GT0 for ground triplet, degeneracy = 1)
c. the triplet *M*_*S*_ = ±1 GS (labeled GT1, degeneracy = 2)
d. the singlet CTs (labeled CT, degeneracy = 2)
e. the singlet LEs (labeled ES for excited singlet, degeneracy = 2)
f. the triplet *M*_*S*_ = 0 LEs (labeled ET0 for excited triplet, degeneracy = 2)
g. the triplet *M*_*S*_ = ±1 LEs (labeled ET1, degeneracy = 4)

**Figure 4:**
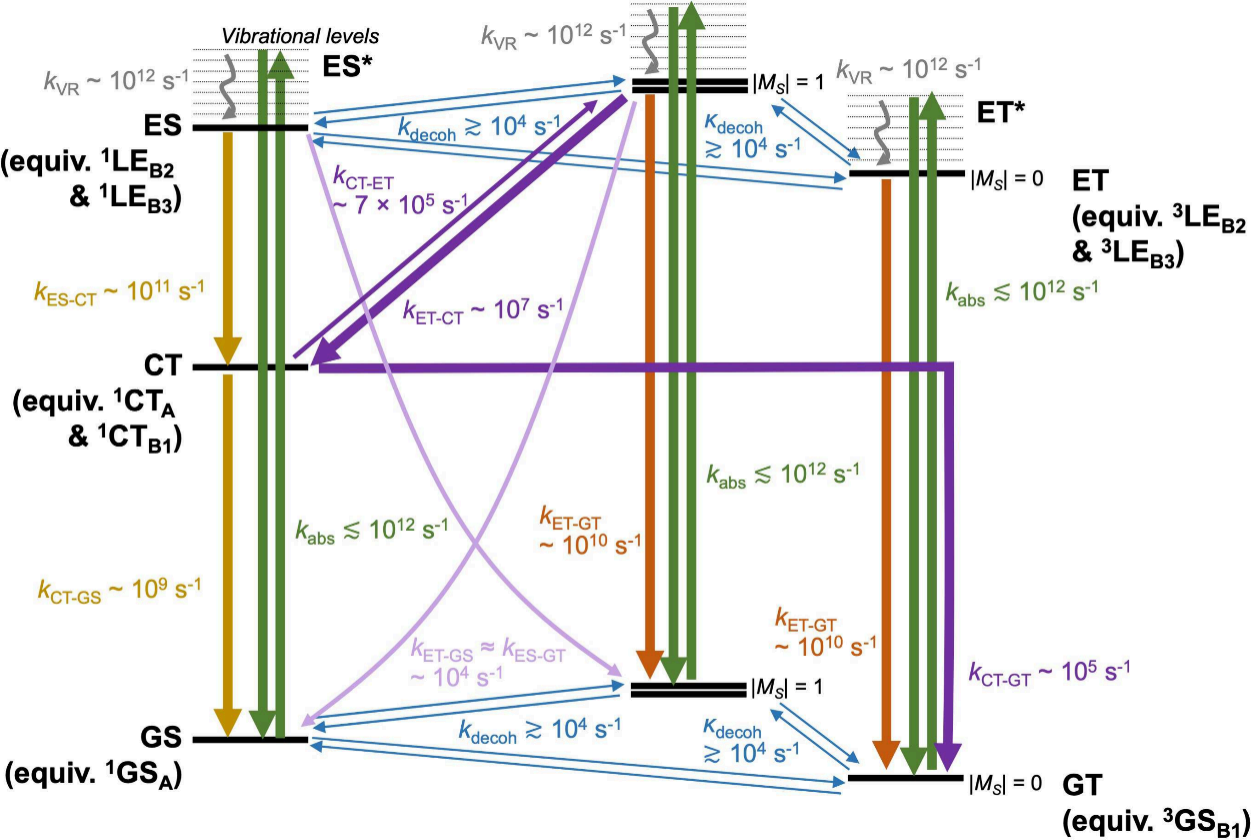
Schematic diagram depicting the excited-state transitions being considered by our microkinetic model.

Here, we continue to assume a spin quantisation axis parallel to the dimer linkage. The kinetic processes connecting these eigenstates and their associated rate constants are presented in Figure 4; there, the symbols adopt their usual meanings. As far as possible, model parameters were selected within experimental reason. Listed below are our justifications for these parameters and the reader may safely skip it at the first pass.

1. Also modeled are the vibrationally excited LE states, labeled with an asterisk (*). These states represent the wave function following an initial Franck–Condon photoexcitation and will, within picoseconds, undergo vibrational relaxation to the ground vibrational level of the respective LE states, labeled without an asterisk ( ).
2. The experimentally observed IC rates from LE to GS triplets are between 10^8^ and 10^10^ s^–1^.^53,54,68,75^ In our model, we shall pick the upper bound as a conservative estimate. Then, because experiments predict the IC rate from CT to GS singlets to be an order of magnitude slower,^53,54^ we place its value at 10^9^ s^–1^. Finally, IC from LE to CT singlets must be faster in view of the smaller energy gap^90^ and we estimate this rate to be 10^11^ s^–1^.
3. The ISC rates are chosen to represent typical organic systems, i.e., around 10^7^ s^–1^ between excited states (i.e., triplet LEs to singlet CTs)^96^ and 10^5^ s^–1^ for relaxation to the ground (i.e., singlet CTs to triplet GSs).^47,48,96^ Because the energy gaps between LEs and GSs are larger than between CTs and GSs, we expect their ISC rates to be scaled down by an order of magnitude to around 10^4^ s^–1^ (this is also expected by the El-Sayed rules; see refs (52 and 54)). Lastly, because the singlet CTs and triplet LEs are relatively close in energy (≳0.05 eV),^52−54^ reverse ISCs are probable with a delayed time scale. By detailed balance, this rate will be around 7 × 10^5^ s^–1^ if we make a safe assumption of a 0.02 eV energy gap at 85 K – these parameters also produce similar ODMR contrasts as experiments^53,54^ when the CT-to-GS ISC channel is shut (more to follow).
4. The weakly coupled pair of doublet spins in the GS can mix and decohere which, in the singlet–triplet basis, translates into population transfers among the singlet and triplet GSs. Therefore, we shall assume a uniform rate constant *k*_decoh_ for mixing between the four near-degenerate magnetic levels (one from the singlet and three from the triplet). This is consistent with Kopp et al.,^54^ where the singlet to triplet GS ISCs occur at around the same time scale as the triplet spin–lattice and spin–spin relaxations. The same decoherence pathway is also expected among the singlet and triplet LEs. Special attention is given to mixing among the triplet spin sublevels, denoted by a rate constant κ_decoh_. In our simulations, we will always set κ_decoh_ = *k*_decoh_ unless a microwave drive is applied (necessary for finding the ODMR contrast). This introduces additional triplet spin mixing that, assuming a saturating microwave field, is mathematically equivalent to the limit of κ_decoh_ → *∞*, i.e., full mixing between the triplet sublevels.^26^

Solving the kinetic model for the steady-state populations yields the ODMR contrast and optically induced spin polarization via the following expressions:^11−13^
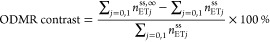
9and
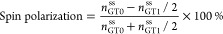
10where the factor of 1/2 arises from the double degeneracy of the triplet *M*_*S*_ = ±1 GSs. Here,  denotes the steady-state population of state Ψ at κ_decoh_ = *k*_decoh_, while  denotes the same quantity at κ_decoh_ → ∞, i.e., under a microwave resonance. (The rate equations are available in Supporting Information 1.)

**Figure 5:**
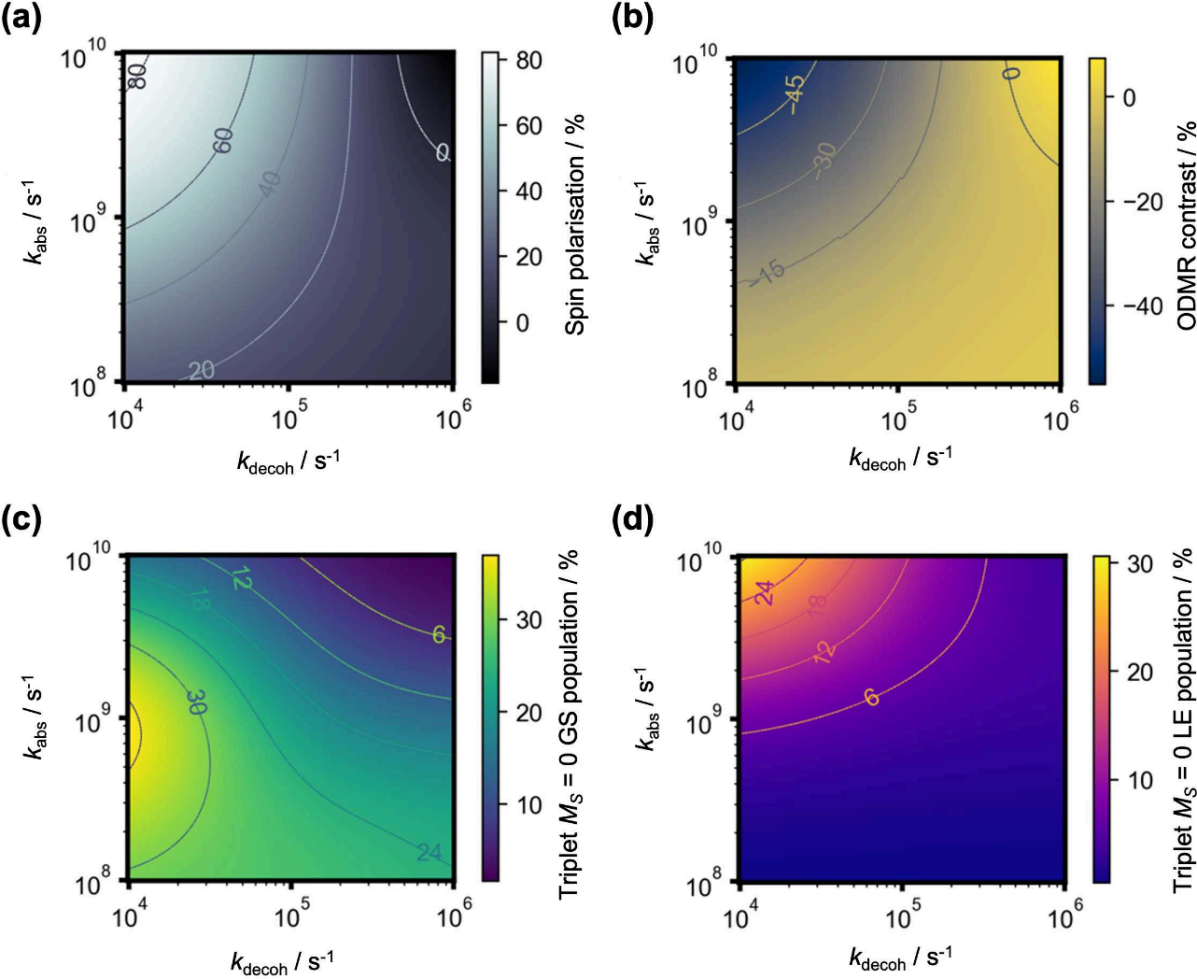
Steady-state solutions to the (a) optically induced spin polarization, (b) ODMR contrast, (c) triplet *M*_*S*_ = 0 GS population, and (d) triplet *M*_*S*_ = 0 LE population (which simulates the emission intensity). Plots are made across varying optical pump rates *k*_abs_ and spin decoherence rates *k*_decoh_ within experimental ranges. Other parameters are defined in Figure 4, chosen to be as realistic as possible.

The results are plotted in Figure 5 across experimental ranges of *k*_abs_ and *k*_decoh_. The former represents the optical pump rate and has an experimental upper bound of 10^12^ s^–1^ for our system (see Supporting Information 2 for estimates of *k*_abs_, which is based on a laser used in teaching laboratories^97^). Meanwhile, the latter denotes the spin relaxation rate, the slowest of which is around 10^4^ s^–1^ at temperatures of around 100 K with faster rates expected at higher temperatures.^45,53,54,69^ Strikingly, at *k*_abs_ = 10^9^ s^–1^ and *k*_decoh_ = 10^4^ s^–1^, a high ODMR contrast of −27.29% is observed with an optically induced spin polarization of 62.76%. The triplet *M*_*S*_ = 0 LE population, which is proportional to the photoluminescence intensity, is also appreciable at 7.34%. Importantly, the proportion of molecules in the triplet GSs is above 25%, suggesting that the observed polarization pathway goes beyond the shelving mechanism described by previous studies.^52−54^ Finally, the above ODMR contrast was obtained at a microwave drive rate of κ_decoh_ ∼ 10^6^ s^–1^, which is experimentally reasonable.^11−13^ For comparison with our earlier theoretical study,^52^ the predicted ODMR contrast in the absence of CT-to-GS ISC is only −0.24%, in agreement with experimental setups that employ the shelving mechanism^53,54^ (this final result was obtained with *k*_CT-GT_ = 0).

## Conclusion

By covalently tethering two benzylic radicals at their *para* positions, the resulting diradical of *D*_2_ point group symmetry has a triplet GS of irrep *B*_1_, lowest singlet CTs of irreps *A* and *B*_1_, and lowest triplet LEs of irreps *B*_2_ and *B*_3_. Thus, by group-theoretical considerations, spin polarization can be achieved via a Δ*M*_*S*_ = ±1 ISC from LEs to CTs, followed by a Δ*M*_*S*_ = 0 ISC from CTs to GSs, just like in an NV center. Moreover, the associated SOC matrix elements are appreciable due to next-nearest-neighbor effects for the former and nearest-neighbor effects for the latter. We expect this success to be generalizable to any diradical based upon two *para*-connected benzylic radicals. This is illustrated in the PT_2_TM-*p*-PT_2_TM diradical: First, the benzylic radicals are *meta*-chlorinated to lock the inter-radical torsion by steric repulsion, ensuring that the GS comprises both triplets and singlets at near-degeneracy. Next, the stabilizing phenyl rings are specially chosen to not break the *C*_2_ rotational symmetry down the benzylic *ipso*-*para* direction, which is the essential symmetry element of the aforementioned benzylic *para*-dimer. Finally, every benzylic radical has one more chloro substituent than each of its phenyl branches, thereby localizing the lowest-lying excitations on the tetra-chlorinated benzylic fragment. Indeed, these expectations are reflected in the ab initio results by producing the desired electronic structure for ODMR.

To our best knowledge, our new design has checked most boxes for a robust optical-spin interface.^17^ Perhaps a minor problem not addressed by this work is the poor emissive properties of our PT_2_TM-*p*-PT_2_TM prototype due to its alternacy symmetry.^52,98,99^ For that, we expect techniques of alternacy symmetry breaking^45,98,99^ and excited-state symmetry breaking^60,61^ employed by the organic light-emitting diode (OLED) community to be useful. In fact, the latter was recently demonstrated by Chowdhury et al.,^53^ who synthesized trityl-based diradicals of near-unity luminescence quantum yields by mesitylating at the *para* positions. When applied to PT_2_TM-*p*-PT_2_TM, a possible diradical would look like Figure 1c, which is a suitable synthetic starting point. Notably, the ODMR contrast for this novel spin polarization pathway is expected to be around 30% with more than 25% of the population being in the triplet manifold at steady state, an unequivocal improvement from earlier theoretical^52^ and experimental^53,54^ works. Regarding qubit operations, our design also overcomes problems of Zeeman-induced spin decoherence and observations of multiple Rabi frequencies during EPR measurements of weakly coupled diradicals. These are all crucial steps toward the realization of optically addressable molecular spin qubits.

## Methods

The geometry of benzyl-*p*-benzyl was relaxed as a triplet on UB3LYP-D3BJ/def2-SVP^100,101^ while enforcing *D*_2*d*_ point group symmetry. Due to the lack of steric hindrances, the optimization converged to a saddle point on the potential energy surface with a single imaginary frequency of amplitude 57.8 cm^–1^. These calculations were done using the ORCA 5.0 code.^102^ We then computed the SOC matrix elements between carbon 2*p* AOs of opposite π-systems using the single-electron form of the SOC operator,^103^ taking each AO to be singly occupied and using a carbon effective nuclear charge of 3.9.^104^ These integrals were computed using PySCF 2.5.0^105^ and the carbon 2*p* AOs were represented by the STO-6G basis set.

Hereafter, all ab initio calculations were performed with the ORCA 5.0 code.^102^ Geometries of PT_2_TM-*p*-PT_2_TM and its central tetra-chlorinated fragment (labeled Cl_4_M-*p*-Cl_4_M) were optimized as triplets on UB3LYP-D3BJ/def2-SVP^100,101^ with harmonic vibrational frequency analyses done to ensure no imaginary frequencies. The resulting geometries had at least *D*_2_ point group symmetries. For the full PT_2_TM-*p*-PT_2_TM diradical, its electronic structure was estimated by spin-unrestricted TDDFT/TDA at the UB3LYP/def2-SVPD level using both triplet and BS singlet ground states as reference. In cases where the latter approach yielded BS solutions of ⟨**S**^2^⟩ ≈ 1, we assumed negligible discrepancies to the singlet energies from spin contamination because the results were similar to the triplet excitations (within 0.1 eV), which are the most-probable spin contaminants.^106^ Excited state irreps were then inferred from the polarizations of the respective transition dipoles. In performing a BS-DFT calculation, we had also obtained the open-shell singlet GS energy via the following expression from Yamaguchi et al.^106^
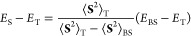
11where *E*_T_, *E*_BS_, and *E*_S_ are the energies of the triplet, BS singlet, and open-shell singlet states while ⟨**S**^2^⟩_T_ and ⟨**S**^2^⟩_BS_ are the respective expectations of the total spin-squared. The smaller Cl_4_M-*p*-Cl_4_M fragment was then used to compute the SOC matrix elements at the MCSCF/CI level, also using the def2-SVPD basis set. For that, we first performed a complete active space self-consistent field (CASSCF) calculation with 10 active electrons and 10 active orbitals, state-averaging the energy over the ground triplet and singlet states (i.e., SA2). Thereafter, the excited states were estimated using the complete active space configuration interaction (CASCI) method, among which the SOC matrix elements were computed. In this work, the electron spin states are expressed in the symmetry-aligned coordinate frame shown in Figure 2e. Finally, with the monoradical of PT_2_TM-*p*-PT_2_TM (labeled PT_2_TM-H), its geometry was first optimized as a doublet on UB3LYP-D3BJ/def2-SVP^100,101^ and the presence of a local minimum of *C*_2_ point group symmetry was confirmed by harmonic vibrational frequency analysis. Then, using the def2-SVPD basis set, orbitals were optimized by CASSCF(11,11) state-specific to the ground doublet, following which the excited doublet energies were obtained by CASCI. In this last calculation, the RIJCOSX approximation (RIJCOSX = resolution of identity approximation for the Coulomb term and chain-of-spheres approximation for the exchange term) was applied using the def2/JK auxiliary basis set. All CASCI energies were corrected by the strongly contracted second-order N-electron valence state perturbation theory (SC-NEVPT2).

## Supporting Information Available

Derivation of analytical results and data from ab initio calculations (PDF)

## Footnotes

1 The El-Sayed rule states that the SOC matrix elements are the largest when the transitioning orbitals adopt different orientations.^79^
