# Supplementary material for: Enhancing the Optically Detected Magnetic Resonance Signal of Organic Molecular Qubits
Source: ACS Cent Sci. 2025 Jan 3;11(1):116–26. doi: 10.1021/acscentsci.4c01632 (PMC11758272; doi:10.1021/acscentsci.4c01632)
Supplement: Supplementary file 1 — oc4c01632_si_001.pdf [file oc4c01632_si_001.pdf]

Supplementary Information for

Enhancing the ODMR Signal of Organic Molecular Qubits

Yong Rui Poh,[1,*] and Joel Yuen-Zhou[1,*]

[1]Department of Chemistry and Biochemistry, University of California San Diego, La Jolla, California 92093, USA
*Corresponding authors. Email addresses: ypoh@ucsd.edu (Y.R.P.); joelyuen@ucsd.edu (J.Y.-Z.)

Contents

S1 Rate equations for the microkinetic model **2**

S2 Estimate of the maximum optical pump rate **2**

S3 Results from ab initio calculations **3**

S3.1 Benzyl-*p*-benzyl diradical 3

S3.2 PT_2TM-*p*-PT_2TM diradical 3

S3.3 Cl_4M-*p*-Cl_4M diradical 8

S3.4 PT_2TM-H monoradical 10

S1 Rate equations for the microkinetic model

The rate equations are

$$\frac{d}{dt}\mathbf{N} = \mathbf{K}\mathbf{N}, \tag{S1}$$

where

$$\mathbf{N} \equiv \begin{pmatrix} n_{\mathrm{GS}} & n_{\mathrm{GT0}} & n_{\mathrm{GT1}} & n_{\mathrm{CT}} & n_{\mathrm{ES}} & n_{\mathrm{ES*}} & n_{\mathrm{ET0}} & n_{\mathrm{ET1}} & n_{\mathrm{ET*0}} & n_{\mathrm{ET*1}} \end{pmatrix}^{\mathrm{T}} \tag{S2}$$

is a column vector containing the eigenstate populations, and

$$\mathbf{K} \equiv \mathrm{diag} \begin{pmatrix} -2k_{\mathrm{abs}} - 3k_{\mathrm{decoh}} \\ -2k_{\mathrm{abs}} - k_{\mathrm{decoh}} - 2\kappa_{\mathrm{decoh}} \\ -4k_{\mathrm{abs}} - k_{\mathrm{decoh}} - \kappa_{\mathrm{decoh}} \\ -k_{\mathrm{CT\text{-}GS}} - 4k_{\mathrm{CT\text{-}ET}} - k_{\mathrm{CT\text{-}GT}}/2 \\ -2k_{\mathrm{ES\text{-}CT}} - 2k_{\mathrm{ES\text{-}GT}} - 6k_{\mathrm{decoh}} \\ -2k_{\mathrm{VR}} - k_{\mathrm{abs}} \\ -k_{\mathrm{ET\text{-}GT}} - 2k_{\mathrm{decoh}} - 4\kappa_{\mathrm{decoh}} \\ -2k_{\mathrm{ET\text{-}CT}} - k_{\mathrm{ET\text{-}GS}} - 2k_{\mathrm{ET\text{-}GT}} - 2k_{\mathrm{decoh}} - 2\kappa_{\mathrm{decoh}} \\ -2k_{\mathrm{VR}} - k_{\mathrm{abs}} \\ -4k_{\mathrm{VR}} - 2k_{\mathrm{abs}} \end{pmatrix}$$

$$+ \begin{pmatrix} 0 & k_{\mathrm{decoh}} & k_{\mathrm{decoh}} & k_{\mathrm{CT\text{-}GS}} & 0 & k_{\mathrm{abs}} & 0 & k_{\mathrm{ET\text{-}GS}} & 0 & 0 \\ k_{\mathrm{decoh}} & 0 & \kappa_{\mathrm{decoh}} & k_{\mathrm{CT\text{-}GT}}/2 & 0 & 0 & k_{\mathrm{ET\text{-}GT}} & 0 & k_{\mathrm{abs}} & 0 \\ 2k_{\mathrm{decoh}} & 2\kappa_{\mathrm{decoh}} & 0 & 0 & 2k_{\mathrm{ES\text{-}GT}} & 0 & 0 & 2k_{\mathrm{ET\text{-}GT}} & 0 & 2k_{\mathrm{abs}} \\ 0 & 0 & 0 & 0 & 2k_{\mathrm{ES\text{-}CT}} & 0 & 0 & 2k_{\mathrm{ET\text{-}CT}} & 0 & 0 \\ 0 & 0 & 0 & 0 & 0 & 2k_{\mathrm{VR}} & 2k_{\mathrm{decoh}} & 2k_{\mathrm{decoh}} & 0 & 0 \\ 2k_{\mathrm{abs}} & 0 & 0 & 0 & 0 & 0 & 0 & 0 & 0 & 0 \\ 0 & 0 & 0 & 0 & 2k_{\mathrm{decoh}} & 0 & 0 & 2\kappa_{\mathrm{decoh}} & 2k_{\mathrm{VR}} & 0 \\ 0 & 0 & 0 & 4k_{\mathrm{CT\text{-}ET}} & 4k_{\mathrm{decoh}} & 0 & 4\kappa_{\mathrm{decoh}} & 0 & 0 & 4k_{\mathrm{VR}} \\ 0 & 2k_{\mathrm{abs}} & 0 & 0 & 0 & 0 & 0 & 0 & 0 & 0 \\ 0 & 0 & 4k_{\mathrm{abs}} & 0 & 0 & 0 & 0 & 0 & 0 & 0 \end{pmatrix} \tag{S3}$$

is a matrix containing the rate constants. Note the factor of 1/2 accompanying $k_{\mathrm{CT\text{-}GT}}$, which is needed because this ISC occurs only from the $^1\mathrm{CT}_A$ state. Also, we have defined the diag operator by

$$\mathrm{diag}\ (a_1, a_2, \cdots, a_j, \cdots)^{\mathrm{T}} \equiv \begin{pmatrix} a_1 & & & & \\ & a_2 & & & \\ & & \ddots & & \\ & & & a_j & \\ & & & & \ddots \end{pmatrix}. \tag{S4}$$

S2 Estimate of the maximum optical pump rate

We estimate the pump rate k_{abs} using the Einstein B coefficient to be

$$k_{\mathrm{abs}} = \frac{e^2 \rho(\nu)}{4\epsilon_0 m_{\mathrm{e}} h\nu} f_{\mathrm{osc}}, \tag{S5}$$

where ν and f_{osc} are the frequency and oscillator strength of the transition in question, and $\rho(\nu)$ is the spectral energy density of the radiation field at frequency ν. The remaining symbols are constants with e for the elementary charge, ϵ_0 for the vacuum permittivity, m_{e} for the electron mass, and h for the Planck constant. To obtain $\rho(\nu)$, we considered a teaching lab set-up [1], which focused a 532 nm Thorlabs DJ532-40 laser with a power P of 40 mW onto a diameter $2r$ of 10 μm. We further assumed the lineshape to be flat around the peak frequency and zero elsewhere; the linewidth $\delta\nu$ was estimated from the user manual to be around 30 MHz. Then, a crude estimate of $\rho(\nu)$ would be

$$\rho(\nu) \approx \frac{P}{c\pi r^2 \delta\nu} = 5.67 \times 10^{-8}\,\mathrm{J\,m^{-3}\,Hz^{-1}}, \tag{S6}$$

where c is the speed of light. Taking ν to be the frequency corresponding to 532 nm wavelength light, we obtain the following upper bound to the pump rate:

$$k_{\mathrm{abs}} \lesssim \left(1.21 \times 10^{14}\,\mathrm{s}^{-1}\right) f_{\mathrm{osc}}. \tag{S7}$$

f_{osc} for the lowest-lying excitation of trityl-based radicals is typically around 0.01 [2], hence

$$k_{\mathrm{abs}} \lesssim 10^{12}\,\mathrm{s}^{-1}. \tag{S8}$$

S3 Results from ab initio calculations

S3.1 Benzyl-*p*-benzyl diradical

The triplet SCF calculation converged to an energy of −14692.84714 eV and an $\langle \mathbf{S}^2 \rangle$ value of 2.066128. The level of theory was UB3LYP-D3BJ/def2-SVP.

Relaxed geometry

```
C          0.00000      -0.00000       0.74467
C          0.85600       0.85600       1.46798
C          0.86047       0.86047       2.85451
C          0.00000      -0.00000       3.60202
C         -0.86047      -0.86047       2.85451
C         -0.85600      -0.85600       1.46798
C          0.00000      -0.00000       5.00984
C          0.00000       0.00000      -0.74467
C         -0.85600       0.85600      -1.46798
C         -0.86047       0.86047      -2.85451
C          0.00000       0.00000      -3.60202
C          0.86047      -0.86047      -2.85451
C          0.85600      -0.85600      -1.46798
C          0.00000       0.00000      -5.00984
H          1.53268       1.53268       3.39476
H         -1.53268      -1.53268       3.39476
H          1.52419       1.52419       0.91856
H         -1.52419      -1.52419       0.91856
H          0.66239       0.66239       5.57165
H         -0.66239      -0.66239       5.57165
H         -1.52419       1.52419      -0.91856
H         -1.53268       1.53268      -3.39476
H          1.53268      -1.53268      -3.39476
H          1.52419      -1.52419      -0.91856
H          0.66239      -0.66239      -5.57165
H         -0.66239       0.66239      -5.57165
```

S3.2 $\mathbf{PT_2TM}$-*p*-$\mathbf{PT_2TM}$ diradical

At the UB3LYP-D3BJ/def2-SVP level, the triplet SCF calculation converged to an energy of −289833.13523 eV and an $\langle \mathbf{S}^2 \rangle$ value of 2.042099. At the UB3LYP/def2-SVPD level, the triplet SCF calculation converged

to an energy of −289830.85584 eV and an $\langle \mathbf{S}^2 \rangle$ value of 2.042475, while the BS SCF calculation converged to an energy of −289830.85637 eV and an $\langle \mathbf{S}^2 \rangle$ value of 1.043240.

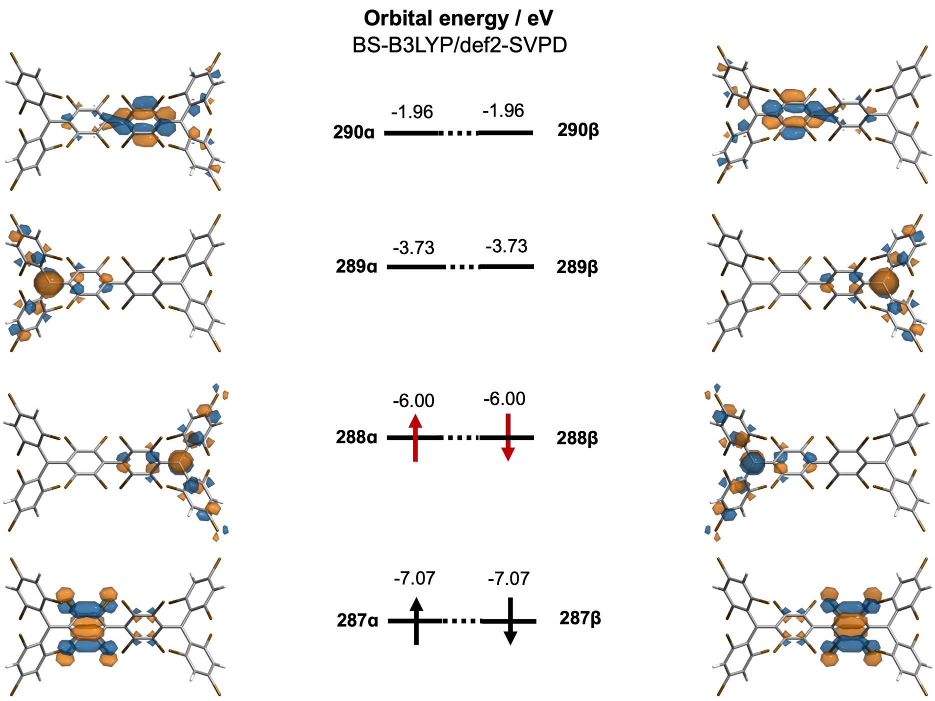

FIG. S1: Frontier MOs (isovalue = 0.040) of PT_2TM-p-PT_2TM in the open-shell BS ground state, calculated at the BS-B3LYP/def2-SVPD level.

Optimised geometry

C	-0.00000574198939	0.00001886283277	-0.74318861439021
C	-0.82678095278225	-0.88014609927434	-1.45536400066426
Cl	-1.86258482682735	-1.95194106627830	-0.56444113784991
C	-0.83158350247726	-0.88052339029383	-2.85590319631644
Cl	-1.95524855748238	-1.91142816496441	-3.68705975754690
C	-0.00000581608866	0.00001070235900	-3.58709128147569
C	-0.00000299485762	0.00000270365669	-5.06000657824186
C	0.83157078934871	0.88055023033315	-2.85590840237393
Cl	1.95523778087506	1.91144805135712	-3.68707097425392
C	0.82676835945782	0.88018086804408	-1.45536905544954
Cl	1.86257456143681	1.95197859168642	-0.56445230199539
C	-0.00000217001731	0.00001886023804	0.74318883337673
C	-0.82677472934388	0.88018224539217	1.45536943291133
Cl	-1.86258005949903	1.95198119702163	0.56445301708662
C	-0.83157635775357	0.88055200382982	2.85590876820934
Cl	-1.95524159458849	1.91145152361032	3.68707165578374
C	-0.00000037868010	0.00001166522908	3.58709138684470
C	-0.00000217031194	0.00000400182907	5.06000668839507
C	0.83157573883127	-0.88052373037234	2.85590315107515
Cl	1.95523992781849	-1.91142956821651	3.68705956373061
C	0.82677228466328	-0.88014698607087	1.45536398549090
Cl	1.86257439566614	-1.95194342327608	0.56444084701128
C	0.17900551702102	1.26105603892104	5.78366664773472

```
C   1.18654999229765      2.19790973989786   5.43741456569588
Cl  2.34674782212454      1.83644445241497   4.18400725246410
C   1.35578328747814      3.40684333867228   6.10821255653891
C   0.50637407962282      3.71770775388578   7.17018267287389
Cl  0.70601325535163      5.22457207778832   8.01770385285598
C   -0.50173341875719      2.83484933278354    7.55989016889500
C   -0.65389520593741      1.63504372449640    6.86985291974398
Cl  -1.99962191524371      0.62889321298102    7.34263264520852
C   -0.17900731381876     -1.26105719724744    5.78365132901835
C   0.65389603742513     -1.63505770789699   6.86983115658126
Cl  1.99962002055447     -0.62890949357922   7.34262357276859
C   0.50173985908195     -2.83487427268144   7.55985059808921
C   -0.50636443216016     -3.71773102480600    7.17013096038665
Cl  -0.70599589518047     -5.22460914677200    8.01762938869358
C   -1.35577635243562     -3.40685394272176    6.10816672444608
C   -1.18654882214552     -2.19790949292590    5.43738678478131
Cl  -2.34674901230920     -1.83643049144512    4.18398555581708
C   -0.17900758247265      1.26105489983025     -5.78366697668087
C   0.65389622129251      1.63504114729646     -6.86985133538927
Cl  1.99962136494639      0.62888751881681     -7.34262894868841
C   0.50173892567358      2.83484762634097     -7.55988803632161
C   -0.50636691203772      3.71770851048111     -7.17018180219864
Cl  -0.70599985874512      5.22457422381186     -8.01770195128842
C   -1.35577896714643      3.40684562968217     -6.10821351254259
C   -1.18655038448497      2.19791090446352     -5.43741629119332
Cl  -2.34675149392943      1.83644779362864     -4.18401136732756
C   0.17900538028328     -1.26105804288897     -5.78365098915979
C   1.18655104625317     -2.19790638119409     -5.43738774456401
Cl  2.34675168860653     -1.83642266135838     -4.18398831465705
C   1.35578318435328     -3.40684960143919     -6.10816867440069
C   0.50637160709494     -3.71772954726441     -7.17013232856319
Cl  0.70600875240596     -5.22460635065789     -8.01763178908284
C   -0.50173704243424     -2.83487703443063     -7.55985028577585
C   -0.65389753780493     -1.63506141783068     -6.86983015992414
Cl  -1.99962641341773     -0.62891874914231     -7.34262045883576
H   2.15383251571159      4.08730443443524   5.81464959278671
H   -1.17756531411163      3.08516224915109    8.37639864788383
H   1.17757398010457     -3.08519688520980   8.37635426492385
H   -2.15382312551000     -4.08731387719391    5.81459441988635
H   1.17757314063382      3.08515940411222    -8.37639495110400
H   -2.15382680646866      4.08730873330317     -5.81465144967596
H   2.15383311378736     -4.08730637419219    -5.81459757501016
H   -1.17757097095111     -3.08520213298905     -8.37635336504712
```

Triplet excited states from UB3LYP/TDDFT/TDA/def2-SVPD

```
STATE  1:  E=   0.093149 au  2.535 eV    20443.8 cm**-1 <S**2> =   2.128548
   282b -> 288b  :     0.057155 (c=  0.23907088)
   283b -> 287b  :     0.083378 (c=  0.28875206)
   285b -> 288b  :     0.348725 (c= -0.59052963)
   286b -> 287b  :     0.457583 (c=  0.67644853)
STATE  2:  E=   0.093205 au  2.536 eV    20456.0 cm**-1 <S**2> =   2.128185
```

```
   282b -> 287b  :     0.063197 (c= -0.25138950)
   283b -> 288b  :     0.083895 (c= -0.28964616)
   285b -> 287b  :     0.397785 (c=  0.63070190)
   286b -> 288b  :     0.400775 (c= -0.63306814)
STATE  3:  E=   0.096181 au  2.617 eV    21109.3 cm**-1 <S**2> =   2.169460
   287a -> 290a  :     0.021460 (c= -0.14649167)
   288a -> 289a  :     0.028774 (c=  0.16962792)
   277b -> 288b  :     0.037751 (c=  0.19429633)
   281b -> 288b  :     0.335959 (c=  0.57961952)
   284b -> 287b  :     0.520854 (c= -0.72170183)
STATE  4:  E=   0.096859 au  2.636 eV    21258.1 cm**-1 <S**2> =   2.173917
   287a -> 289a  :     0.038217 (c=  0.19549194)
   288a -> 290a  :     0.035136 (c= -0.18744546)
   277b -> 287b  :     0.033009 (c= -0.18168410)
   281b -> 287b  :     0.373787 (c= -0.61138117)
   284b -> 288b  :     0.465990 (c=  0.68263493)
STATE  5:  E=   0.096945 au  2.638 eV    21276.9 cm**-1 <S**2> =   2.150603
   287a -> 292a  :     0.028892 (c=  0.16997656)
   288a -> 291a  :     0.024328 (c= -0.15597515)
   282b -> 288b  :     0.351610 (c= -0.59296688)
   283b -> 287b  :     0.375825 (c= -0.61304560)
   285b -> 288b  :     0.035408 (c= -0.18816884)
   286b -> 287b  :     0.133565 (c=  0.36546515)
STATE  6:  E=   0.097079 au  2.642 eV    21306.4 cm**-1 <S**2> =   2.150283
   287a -> 291a  :     0.024191 (c= -0.15553573)
   288a -> 292a  :     0.030420 (c=  0.17441401)
   282b -> 287b  :     0.378789 (c=  0.61545844)
   283b -> 288b  :     0.344036 (c=  0.58654606)
   285b -> 287b  :     0.054106 (c=  0.23260648)
   286b -> 288b  :     0.119298 (c= -0.34539516)
STATE  7:  E=   0.104197 au  2.835 eV    22868.6 cm**-1 <S**2> =   2.125107
   287a -> 291a  :     0.012208 (c=  0.11049073)
   288a -> 293a  :     0.012435 (c= -0.11151433)
   279b -> 287b  :     0.471603 (c= -0.68673332)
   280b -> 288b  :     0.437931 (c= -0.66176332)
   282b -> 287b  :     0.012287 (c=  0.11084545)
STATE  8:  E=   0.104205 au  2.836 eV    22870.3 cm**-1 <S**2> =   2.124973
   287a -> 293a  :     0.012500 (c= -0.11180518)
   288a -> 291a  :     0.012225 (c=  0.11056768)
   279b -> 288b  :     0.434934 (c=  0.65949515)
   280b -> 287b  :     0.474013 (c=  0.68848638)
   282b -> 288b  :     0.011371 (c= -0.10663722)
   283b -> 287b  :     0.011275 (c= -0.10618423)
-----------------------------------------------------------------------------
         ABSORPTION SPECTRUM VIA TRANSITION ELECTRIC DIPOLE MOMENTS
-----------------------------------------------------------------------------
```

State	Energy (cm-1)	Wavelength (nm)	fosc	T2 (au**2)	TX (au)	TY (au)	TZ (au)
1	20443.8	489.1	0.003282776	0.05286	0.22991	-0.00163	-0.00000
2	20456.0	488.9	0.019409525	0.31237	-0.00069	-0.55890	0.00005
3	21109.3	473.7	0.060043138	0.93641	-0.00003	0.00005	-0.96768
4	21258.1	470.4	0.000000020	0.00000	0.00016	-0.00052	-0.00009

5	21276.9	470.0	0.000801065	0.01239	0.11133	0.00083	-0.00019
6	21306.4	469.3	0.061450615	0.94949	-0.00010	0.97442	0.00010
7	22868.6	437.3	0.005164043	0.07434	-0.00696	-0.27257	0.00004
8	22870.3	437.2	0.011462876	0.16501	0.40618	-0.00466	0.00004

Singlet excited states from BS-B3LYP/TDDFT/TDA/def2-SVPD

```
STATE  1:  E=   0.072498 au  1.973 eV    15911.4 cm**-1 <S**2> =   0.182750
   287a -> 288a  :     0.440292 (c=  0.66354522)
   287b -> 288b  :     0.555762 (c= -0.74549461)
STATE  2:  E=   0.072512 au  1.973 eV    15914.6 cm**-1 <S**2> =   0.181942
   287a -> 288a  :     0.555944 (c= -0.74561668)
   287b -> 288b  :     0.440475 (c= -0.66368284)
STATE  3:  E=   0.093217 au  2.537 eV    20458.7 cm**-1 <S**2> =   1.128499
   284a -> 288a  :     0.074182 (c=  0.27236462)
   285a -> 288a  :     0.059299 (c=  0.24351293)
   286a -> 288a  :     0.349276 (c=  0.59099606)
   284b -> 288b  :     0.071064 (c= -0.26657893)
   285b -> 288b  :     0.056877 (c=  0.23848921)
   286b -> 288b  :     0.334941 (c= -0.57874068)
STATE  4:  E=   0.093249 au  2.537 eV    20465.8 cm**-1 <S**2> =   1.129617
   284a -> 288a  :     0.073716 (c=  0.27150602)
   285a -> 288a  :     0.057721 (c=  0.24025234)
   286a -> 288a  :     0.331261 (c=  0.57555284)
   284b -> 288b  :     0.076815 (c=  0.27715504)
   285b -> 288b  :     0.060221 (c= -0.24540067)
   286b -> 288b  :     0.345538 (c=  0.58782483)
STATE  5:  E=   0.096325 au  2.621 eV    21140.9 cm**-1 <S**2> =   1.166153
   278a -> 288a  :     0.012457 (c=  0.11160882)
   281a -> 288a  :     0.078066 (c=  0.27940275)
   283a -> 288a  :     0.352531 (c= -0.59374329)
   287a -> 289a  :     0.020073 (c= -0.14168042)
   278b -> 288b  :     0.012411 (c=  0.11140512)
   281b -> 288b  :     0.078090 (c=  0.27944607)
   283b -> 288b  :     0.351778 (c= -0.59310870)
   287b -> 289b  :     0.020113 (c= -0.14182153)
STATE  6:  E=   0.096800 au  2.634 eV    21245.1 cm**-1 <S**2> =   1.175258
   281a -> 288a  :     0.076101 (c=  0.27586331)
   283a -> 288a  :     0.343482 (c= -0.58607367)
   287a -> 289a  :     0.031310 (c=  0.17694758)
   281b -> 288b  :     0.076389 (c= -0.27638594)
   283b -> 288b  :     0.343945 (c=  0.58646847)
   287b -> 289b  :     0.031262 (c= -0.17681019)
STATE  7:  E=   0.097041 au  2.641 eV    21298.0 cm**-1 <S**2> =   1.150917
   284a -> 288a  :     0.359091 (c= -0.59924219)
   286a -> 288a  :     0.087502 (c=  0.29580779)
   287a -> 291a  :     0.024935 (c=  0.15790760)
   284b -> 288b  :     0.353061 (c= -0.59418932)
   286b -> 288b  :     0.086024 (c=  0.29329775)
   287b -> 291b  :     0.025363 (c= -0.15925665)
STATE  8:  E=   0.097082 au  2.642 eV    21307.0 cm**-1 <S**2> =   1.151071
   284a -> 288a  :     0.354277 (c=  0.59521151)
```

```
   286a -> 288a  :     0.084660 (c= -0.29096448)
   287a -> 291a  :     0.026324 (c=  0.16224807)
   284b -> 288b  :     0.360305 (c= -0.60025406)
   286b -> 288b  :     0.086093 (c=  0.29341546)
   287b -> 291b  :     0.025886 (c=  0.16089062)
STATE  9:  E=   0.104217 au  2.836 eV    22872.9 cm**-1 <S**2> =   1.125907
   280a -> 288a  :     0.511958 (c=  0.71551211)
   284a -> 288a  :     0.012066 (c=  0.10984483)
   280b -> 288b  :     0.397747 (c= -0.63067226)
   287b -> 291b  :     0.011606 (c= -0.10773213)
STATE 10:  E=   0.104226 au  2.836 eV    22875.0 cm**-1 <S**2> =   1.125839
   280a -> 288a  :     0.397434 (c= -0.63042366)
   287a -> 291a  :     0.011375 (c= -0.10665240)
   280b -> 288b  :     0.511638 (c= -0.71528872)
   284b -> 288b  :     0.012492 (c= -0.11176961)
-----------------------------------------------------------------------------
         ABSORPTION SPECTRUM VIA TRANSITION ELECTRIC DIPOLE MOMENTS
-----------------------------------------------------------------------------
```

State	Energy (cm-1)	Wavelength (nm)	fosc	T2 (au**2)	TX (au)	TY (au)	TZ (au)
1	15911.4	628.5	0.000009813	0.00020	-0.00000	0.00000	0.01425
2	15914.6	628.4	0.002904789	0.06009	-0.00000	-0.00000	0.24513
3	20458.7	488.8	0.019443057	0.31287	0.00239	-0.55934	0.00004
4	20465.8	488.6	0.003239369	0.05211	0.22820	0.00583	0.00000
5	21140.9	473.0	0.059563393	0.92754	0.00002	0.00005	0.96309
6	21245.1	470.7	0.000000015	0.00000	-0.00002	0.00028	-0.00040
7	21298.0	469.5	0.000872328	0.01348	0.11605	0.00413	-0.00012
8	21307.0	469.3	0.061700546	0.95333	0.00049	-0.97638	-0.00000
9	22872.9	437.2	0.005190136	0.07470	-0.02564	0.27211	-0.00002
10	22875.0	437.2	0.011509315	0.16564	0.40663	0.01714	0.00004

S3.3 Cl_4M-*p*-Cl_4M diradical

The triplet SCF calculation converged to an energy of −114701.41640 eV and an $\langle \mathbf{S}^2 \rangle$ value of 2.075353. The level of theory was UB3LYP-D3BJ/def2-SVP.

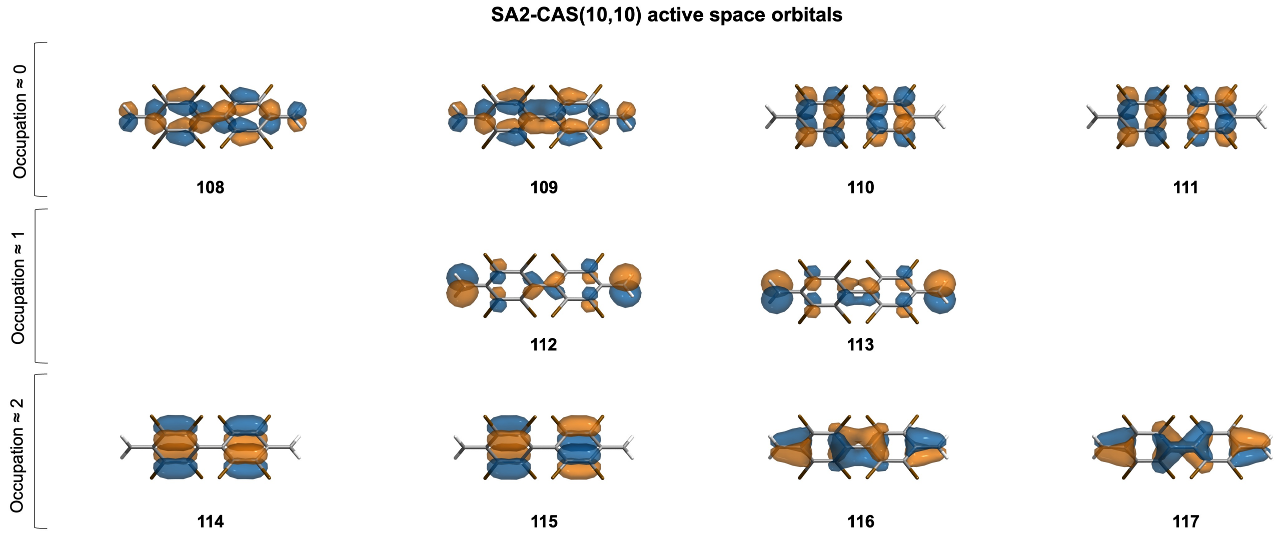

FIG. S2: Active space MOs (isovalue = 0.040) of Cl_4M-*p*-Cl_4M from a CASSCF(10,10)/def2-SVPD calculation state-averaged over the triplet and singlet ground states (i.e. SA2).

Table S1: Excited-state properties of Cl_4M-*p*-Cl_4M, calculated at the SA2-CASSCF(10,10)/CASCI/SC-NEVPT2/def2-SVPD level. The triplet and singlet LE_{B_2} states are dominated by the following transitions: 114 → 113, 115 → 112, 113 → 111, 112 → 110. The triplet and singlet LE_{B_3} states are dominated by the following transitions: 114 → 112, 115 → 113, 112 → 111, 113 → 110.

Triplets		Singlets	
**Energy / eV**	**Assignment**	**Energy / eV**	**Assignment**
0.006	$^3GS_{B_1}$	0.000	^1GS_A
		1.617	^1CT_A
		1.622	$^1CT_{B_1}$
2.604	$^3LE_{B_2}$	2.612	$^1LE_{B_2}$
2.604	$^3LE_{B_3}$	2.612	$^1LE_{B_3}$

Optimised geometry

```
C    0.00000041285253     0.00000029555640    0.74297677529495
C    0.85732573641907     0.85732631288620    1.46036723377016
C    0.86449831678849     0.86449897249083    2.85373875414654
Cl   1.93779307205256     1.93779488416541    3.69549743130807
C   -0.00000014382067    -0.00000005359037    3.61203276227255
C   -0.00000040175185    -0.00000028309773    5.01220248078794
C   -0.86449833501065    -0.86449887424287    2.85373817543095
Cl  -1.93779341609317    -1.93779493750990    3.69549621346067
C   -0.85732522342353    -0.85732588248186    1.46036665514901
Cl   1.90872191143777     1.90872327279931    0.56204835859350
Cl  -1.90872102502467    -1.90872254490025    0.56204699493039
H    0.66403776579042     0.66403836065583    5.56214484145260
H   -0.66403867943697    -0.66403921249331    5.56214434734771
C    0.00000059154361     0.00000047708230   -0.74297680842324
C   -0.85732498072283     0.85732650236958   -1.46036693763725
Cl  -1.90872068612633     1.90872347952667   -0.56204752525952
```

C	-0.86449821272356	0.86449900828024	-2.85373843687065
Cl	-1.93779350464977	1.93779467711227	-3.69549669018358
C	-0.00000016226542	-0.00000015609920	-3.61203278430882
C	-0.00000070594291	-0.00000065316442	-5.01220250752925
C	0.86449845299473	-0.86449884127681	-2.85373851575171
Cl	1.93779309206189	-1.93779501603972	-3.69549697710167
C	0.85732597981825	-0.85732568976200	-1.46036699911219
Cl	1.90872202824070	-1.90872234501100	-0.56204762706680
H	0.66403725680076	-0.66403966557926	-5.56214462005595
H	-0.66403913980843	0.66403791232367	-5.56214459464441

S3.4 PT_2TM-H monoradical

The doublet SCF calculation converged to an energy of −144932.58265 eV and an $\langle \mathbf{S}^2 \rangle$ value of 0.770696. The level of theory was UB3LYP-D3BJ/def2-SVP.

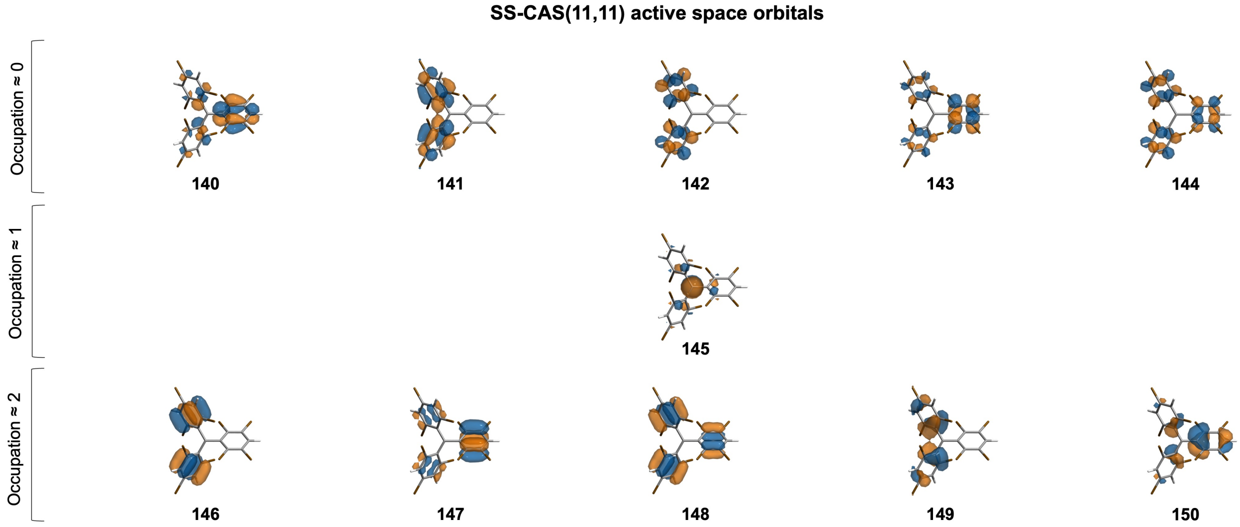

FIG. S3: Active space MOs (isovalue = 0.040) of PT_2TM-H from a CASSCF(11,11)/def2-SVPD calculation state-specific to the ground doublet.

Table S2: Excited-state properties of PT_2TM-H, calculated at the SS-CASSCF(11,11)/CASCI/SC-NEVPT2/def2-SVPD level.

Energy / eV	Main contribution	Assignment
0.000	–	^{2}GS$_B$
2.780	147 → 145	^{2}LE$_A$
2.988	146 → 145	^{2}LE$_B$
3.078	149 → 145	^{2}LE$_A$
3.296	150 → 145	^{2}LE$_B$

Optimised geometry

C	-0.00000100878170	0.00000169583685	4.12761634902078
C	-0.85851151864839	-0.84825523996764	3.42970609427926

Cl	-1.92674366505162	-1.87460275292160	4.33649252936941
C	-0.86593552020095	-0.85043019279702	2.02751198441216
Cl	-2.02880823448355	-1.83842286789924	1.19812103091070
C	0.00000078223341	-0.00000051292764	1.29728867140791
C	0.00000068858206	-0.00000072793933	-0.17626186450116
C	0.86593621128483	0.85043028720999	2.02751182881201
Cl	2.02880918443113	1.83842267932249	1.19812093592387
C	0.85851038281088	0.84825754834784	3.42970593743530
Cl	1.92674044220314	1.87460731220680	4.33649225374461
H	-0.00000190376054	0.00000268662313	5.21685381496298
C	-0.12598996386433	1.26578433548672	-0.90410738379568
C	0.72838903626060	1.60545985538092	-1.98454582181853
Cl	2.04360888115442	0.55206219939831	-2.43980438625745
C	0.62212380120034	2.80591663776188	-2.68244689972772
C	-0.35989860412666	3.72341732904067	-2.30653883135869
Cl	-0.50466198860348	5.23000087113262	-3.16590016424509
C	-1.22733044492931	3.44765896661459	-1.24944541387940
C	-1.10390077055072	2.23733510764173	-0.57129516226888
Cl	-2.28661969547178	1.91766442755174	0.67187747918888
C	0.12599048854006	-1.26578580916702	-0.90410764623741
C	1.10390190812176	-2.23733651837442	-0.57129696102111
Cl	2.28662214563588	-1.91766617759692	0.67187451667678
C	1.22733129011585	-3.44765996512256	-1.24944800338326
C	0.35989862743596	-3.72341798012113	-2.30654085170079
Cl	0.50466181794816	-5.23000100911267	-3.16590313167695
C	-0.62212426204596	-2.80591724030012	-2.68244763865872
C	-0.72838916842485	-1.60546084978066	-1.98454578265692
Cl	-2.04360934075232	-0.55206304737579	-2.43980314450043
H	1.31251887555110	3.02906092760722	-3.49460860037946
H	-2.00486979890244	4.15580546212846	-0.96671010777986
H	2.00487110610469	-4.15580639100197	-0.96671381080138
H	-1.31251978101571	-3.02906104688623	-3.49460909226058

References

[1] H. Zhang, C. Belvin, W. Li, J. Wang, J. Wainwright, R. Berg, and J. Bridger, American Journal of Physics **86**, 225 (2018).

[2] Y. R. Poh, D. Morozov, N. P. Kazmierczak, R. G. Hadt, G. Groenhof, and J. Yuen-Zhou, Journal of the American Chemical Society **146**, 15549 (2024).
